# Supplementary figures and images for: New Male Users of Lipid-Lowering Drugs for Primary Prevention of Cardiovascular Disease: The Impact of Treatment Persistence on Morbimortality. A Longitudinal Study
Source: Int J Environ Res Public Health. 2020 Oct 20;17(20):7653. doi: 10.3390/ijerph17207653 (PMC7593937; doi:10.3390/ijerph17207653)

**Score <2**

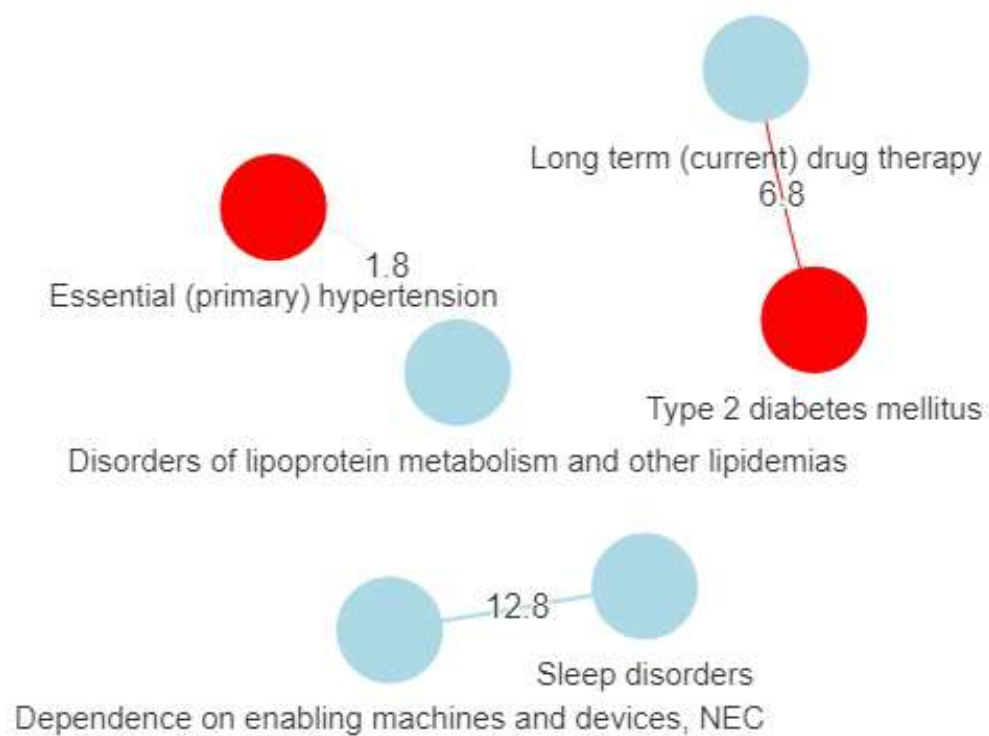

Supplement: Supplementary file 1 [file ijerph-17-07653-s001.zip › Appendix1a.pdf]

**Score  $\geq 2$**

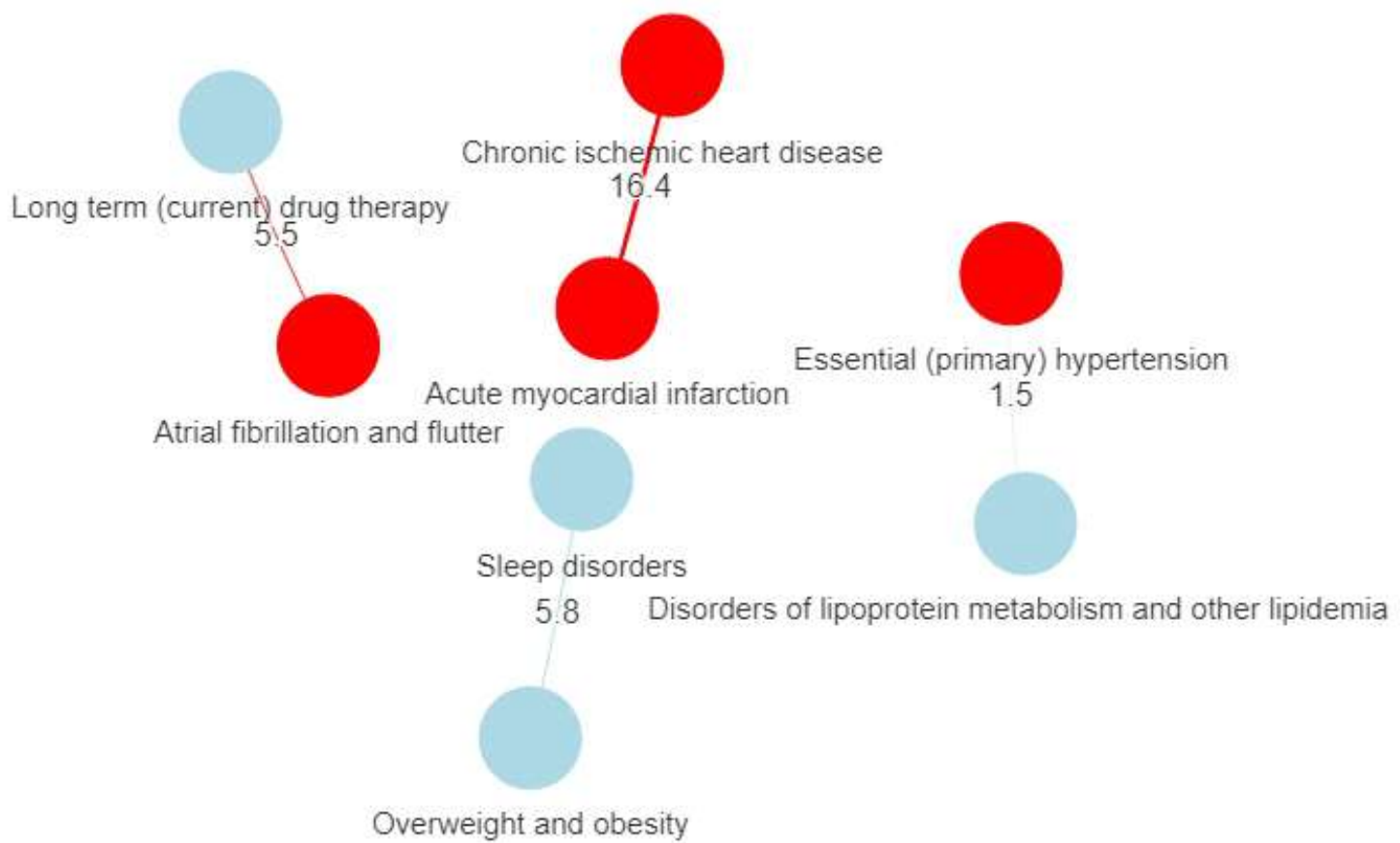

Supplement: Supplementary file 1 [file ijerph-17-07653-s001.zip › Appendix1b.pdf]
